# Genetic testing is necessary for correct diagnosis and treatment in patients with isolated methylmalonic aciduria: a case report

**DOI:** 10.1186/s12887-021-03067-3

**Published:** 2021-12-16

**Authors:** Katarína Brennerová, Martina Škopková, Mária Ostrožlíková, Jana Šaligová, Juraj Staník, Vladimír Bzdúch, Daniela Gašperíková

**Affiliations:** 1 Department of Paediatrics, Medical Faculty of Comenius University and National Institute for Children’s Diseases, Limbová 1, 833 40 Bratislava, Slovakia; 2 Laboratory of Diabetes and Metabolic Disorders, Institute of Experimental Endocrinology, Biomedical Research Center, Slovak Academy of Sciences, Dúbravská cesta 9, 845 05 Bratislava, Slovakia; 3 Department of Laboratory Medicine, National Institute for Children’s Diseases, Limbova 1, 833 40 Bratislava, Slovakia; 4 Department of Paediatrics, Medical Faculty of P. J. Šafárik University in Košice, Trieda SNP 1, 040 11 Košice, Slovakia

**Keywords:** isolated methylmalonic aciduria, cobalamin responsive, genetic testing, case report

## Abstract

**Background:**

Isolated methylmalonic aciduria can be caused by pathogenic mutations in the gene for methylmalonyl-CoA mutase or in the genes encoding enzymes involved in the intracellular metabolism of cobalamin. Some of these mutations may be cobalamin responsive. The type of methylmalonic aciduria cannot always be assumed from clinical manifestation and the responsiveness to cobalamin has to be assessed for appropriate cobalamin administration, or to avoid unnecessary treatment. The cases presented herein highlight the importance of genetic testing in methylmalonic aciduria cases and the need for standardisation of the *in vivo* cobalamin-responsiveness assessment.

**Case presentation:**

We describe two patients who presented in the first week of life with rapid neurological deterioration caused by metabolic acidosis with severe hyperammonaemia requiring extracorporeal elimination in addition to protein restriction, energy support, carnitine, and vitamin B12 treatment. The severity of the clinical symptoms and high methylmalonic acid concentrations in the urine (>30,000 μmol/mmol of creatinine) without hyperhomocysteinaemia in both of our patients suggested isolated methylmalonic aciduria. Based on the neonatal manifestation and the high methylmalonic acid urine levels, we assumed the cobalamin non-responsive form. The *in vivo* test of responsiveness to cobalamin was performed in both patients. Patient 1 was evaluated as non-responsive; thus, intensive treatment with vitamin B12 was not used. Patient 2 was responsive to cobalamin, but the dose was decreased to 1 mg i.m. every two weeks with daily oral treatment due to non-compliance. Genetic tests revealed bi-allelic mutations in the genes *MMAB* and *MMAA* in Patient 1 and 2, respectively. Based on these results, we were able to start intensive treatment with hydroxocobalamin in both patients. After the treatment intensification, there was no acute crisis requiring hospitalisation in Patient 1, and the urine methylmalonic acid levels further decreased in Patient 2.

**Conclusions:**

Despite carrying out the *in vivo* test of responsiveness to cobalamin in both patients, only the results of molecular genetic tests led us to the correct diagnosis and enabled intensive treatment with hydroxocobalamin. The combination of the standardized *in vivo* test of cobalamin responsiveness and genetic testing is needed for accurate diagnosis and appropriate treatment of isolated methylmalonic aciduria.

## Background

Methylmalonic aciduria (MMA) is one of the most common organic acidurias, with an incidence of 1:48,000 to 1:250,000 [1]. Methylmalonyl-CoA (MMCoA) is accumulated in the body as a result of the disrupted degradation of valine, isoleucine, methionine, and threonine [2, 3]. Organic acids, particularly methylmalonate and methylcitrate, are formed from MMCoA in an alternative metabolic pathway and can cause metabolic acidosis, carnitine deficiency, and secondary hyperammonaemia. When the accumulation of methylmalonic acid is not accompanied by a significant elevation of homocysteine in plasma, it is classified as isolated methylmalonic aciduria (iMMA) [4–6].

iMMA can be caused by pathogenic mutations in the *MMUT* gene (MIM#609058) encoding l-methylmalonyl-CoA mutase (*mut* type), in the *MCEE* gene (MIM#608419) encoding methylmalonyl-CoA epimerase, or in the genes encoding enzymes of intracellular metabolism of vitamin B12 (cobalamin, Cbl) leading to the synthesis of MMUT cofactor adenosylcobalamin (AdoCbl): *MMAA*, *MMAB*, and *MMADHC* (*CblA*, *CblB*, and *CblD2* types, respectively) [2, 4, 7]. The *MMAB* gene (MIM#607568) encodes an adenosyltransferase that catalyses the transfer of adenosine from ATP to cobalamin to generate AdoCbl. The *MMAA* gene (MIM#607481) encodes a protein responsible for proper AdoCbl gating and incorporation from MMAB to the l-methylmalonyl-CoA mutase through a GTP-dependent interaction [8]. If the administration of Cbl in high doses increases the activity of MMUT, the condition is described as Cbl-responsive iMMA. iMMA subtypes are diagnosed by enzyme assay analysis and/or molecular studies [5]. Cbl non-responsive forms (*mut*^*0*^ and large proportion of *CblB*) manifest most often in the neonatal period [5]. They usually have a more severe clinical course, with frequent metabolic crises despite treatment [7, 9]. Neurological complications are common. Damage of the basal ganglia leads to disabling movement disorders with choreoathetosis, dystonia, and para/quadriparesis. Chronic progressive renal insufficiency often appears as early as in childhood [10, 11].

Cbl responsive forms (typically *CblA* and *mut*^-^) are characterized by a later manifestation, most often provoked by an acute infection [7, 12], or they develop gradually with symptoms of failure to thrive, hypotonus, and slow motor and mental development. Serious neurological complications and chronic kidney disease are less frequent. However, if Cbl treatment is underestimated, the disease may be complicated by renal failure [10]. The *in vivo* responsiveness to vitamin B12 should be determined in all patients. The test most often used to assess cobalamin responsiveness is urinary methylmalonic acid excretion before and after administration of cobalamin. Methylmalonic acid concentrations in plasma and propionylcarnitine in dried blood are not commonly used [13]. The test has to be done outside of a metabolic crisis, and a significant reduction (more than 50 %) of typical metabolite production indicates sensitivity to Cbl treatment. Hydroxocobalamin (OH-Cbl) is the preferred drug for the test [7, 14–16].

Here, in two case reports, we present the pitfalls of determining the iMMA type based on clinical course, the concentration of MMA in urine, and sensitivity to vitamin B12.

## Case presentations

During the years 2002-2020, 7 patients with iMMA were diagnosed in our centre. All were treated with diet, carnitine, and vitamins. Vitamin B12 treatment was modified according to the result of the *in vivo* Cbl sensitivity test.

Here we report on two patients who manifested similarly in the first week of life with a severe metabolic crisis. Neurological symptomatology was accompanied by metabolic acidosis, hyperammonaemia was above 1,000 μmol/L. Despite similar clinical signs in the manifestation period, the children differed in the course of the disease. Data were obtained retrospectively from the inpatient documentation and outpatient follow-up.

### Patient 1

The first patient with iMMA was considered as *mut*^*0*^ type due to the severe clinical symptomatology. The boy was born in term with a birth weight of 2,900 g and a length of 49 cm. On the third postnatal day, he presented with vomiting, Kussmaul breathing, apathy, and hypotonus. Severe metabolic acidosis (pH<7.2, base excess -23 mmol/L) with hyperammonaemia (260 μmol/L) was confirmed. The profile of organic acids in the urine indicated the diagnosis of MMA - high excretion of methylmalonic acid and methylcitrate. He also had a slightly increased concentration of tHcy (24 μmol/L). The vitamin B12 levels in serum were not assessed as this measurement was not available at that time in our setting. The concentration of ammonia in the blood rose rapidly to 1,097 μmol/L and the child required elimination treatment in addition to the carnitine and low-protein diet. Ammonia in plasma decreased from 1,097 to 64 μmol/l, and methylmalonic acid in the urine decreased from 32,900 to 57 μmol/mmol creatinine within one week under this treatment. In the test of Cbl sensitivity (i.m. administration of 100 μg of cyano-Cbl for 7 days), the tolerance of natural proteins was not increased significantly. The patient was therefore evaluated as non-responsive to Cbl and intensive treatment with vitamin B12 was not used.

**Fig. 1:**
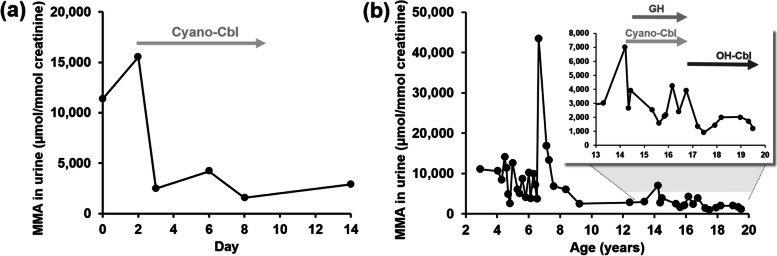
Urinary methylmalonic acid concentration in Patient 1. **(a)** During metabolic decompensation with prolonged metabolic acidosis at the age of 4 years. The child was treated with cyano-Cbl, 1 mg/day i.m. for 7 days. He was also treated with high-dose energy infusions delivered via a central venous catheter. **(b)** During the long-term follow-up. Only data from outpatient check-ups were included

The child’s motor development was adequate during the first year of life. However, within the next three years of life, he overcame several metabolic decompensations with severe metabolic acidosis, hyperammonaemia, and subsequent deterioration in neurological development. At the age of 4 years, during a severe metabolic crisis with prolonged metabolic acidosis, the child was treated with 1 mg/day cyano-Cbl i.m. for 7 days. He had a significant reduction of urinary methylmalonic acid (Fig. 1a). However, he was also treated with high-dose energy infusions delivered via a central venous catheter. Therefore, we stopped the cyano-Cbl since the patient was metabolically compensated.

At the age of 11 years, vitamin B12 deficiency was confirmed by the low total cobalamin concentration in serum (118 pmol/L; normal range 145-637) and the patient had slightly elevated plasma tHcy. We assumed a nutritional vitamin B12 deficiency caused by the low-protein diet with a very small amount of animal proteins. After the replacement of vitamin B12, both tHcy and vitamin B12 levels normalised (vitamin B12 222 pmol/L). We continued with long-term oral substitution and intermittent i.m. doses of cyano-Cbl to prevent vitamin B12 deficiency.

At the age of 14 years, *MMUT* deficiency was ruled out by molecular genetic analysis, and the *CblB* defect in the *MMAB* gene (NM_052845.4*:*c.[556C>T];[556C>T], p.[Arg(R186Trp)];[(Arg186Trp)]) was later confirmed based on whole-exome sequencing. This variant is the most frequent pathogenic variant in the *MMAB* gene in the European population [9, 17] and is classified as pathogenic according to ACMG guidelines [18] with ClinGen Sequence Variant Interpretation Recommendations for PM2 and PM3 (PM3_very strong, PS4, PP3, PM2_sup). As some patients with the *CblB* defect were described as being Cbl-responsive [7], we started with more intensive vitamin B12 treatment. First, we increased the i.m. dose of cyano-Cbl (from 0.3 mg intermittently every two weeks to 1 mg regularly every week), and, after several months, we switched to OH-Cbl (1 mg i.m. every week). The urine methylmalonic acid decreased consequently on the more intensive Cbl treatment (Fig. 1b). The serum concentration of vitamin B12 on this treatment was > 1467 pmol/L (the upper detection limit of the method).

The first signs of renal insufficiency in the patient were detected at the age of 9 years. In addition, growth retardation, with proven growth hormone deficiency was treated with growth hormone (0.017 and later 0.01 mg/kg/day) for a period of two years (until the age of 17 years) (Fig. 1b). Currently, the patient is 19 years old with a moderate intellectual disability, signs of basal ganglia damage on MRI, and chronic kidney disease in stage CKD3. He has not required hospitalization for metabolic decompensation during the last 2 years on the more intensive treatment with vitamin B12. The patient also reported better physical activity tolerance. However, we did not notice amelioration of renal function indicated by glomerular filtration and serum cystatin C levels. The dose of hydroxocobalamin was further increased to 2x1 mg/week at the time this manuscript was finalised.

### Patient 2

The second patient with iMMA was considered as *mut*^-^ or *CblA* type according to her Cbl sensitivity.

The girl was born in term with a birth weight of 2,950 g and a length of 51 cm. On the fourth postnatal day, she developed apathy, hypothermia, and dehydration with metabolic acidosis and hyperammonaemia (1,601 μmol/L). Typical iMMA metabolites were found in the urine. The tHcy and vitamin B12 values in the blood were normal (338 pmol/L). She required peritoneal dialysis for 20 hours together with a low-protein diet and carnitine and vitamin B12 treatment. Ammonaemia and acidosis were normalised within 24 hours from the start of the treatment. OH-Cbl administration 1 mg i.v. every second day for 10 days was used as the *in vivo* Cbl sensitivity test. During this test, the child started to tolerate natural proteins (in a dose of 1.4 g/kg/day) and methylmalonic acid in urine decreased from 47,686 to 164 μmol/mmol creatinine. We concluded that this was the Cbl responsive type of iMMA. During the follow-up, the child was metabolically stable, but she was very negativistic during the i.m. application of cobalamin. Therefore, we reduced the Cbl dose to 1 mg i.m. every two weeks with daily oral treatment (the dose varied from 0.3 mg once a week to 0.5 mg per day). Cyano-Cbl was used in long-term treatment, as OH-Cbl was available in our country only for patients with confirmed intracellular cobalamin defect. The further course of the disease was favourable despite a diet with minimal protein restriction. The serum vitamin B12 levels were increased (834 - >1,476 pmol/L). Metabolic decompensations occurred rarely (3-times during the 8-years of follow-up), and they were always provoked by an acute infection, accompanied by mild metabolic acidosis, mild hyperammonaemia, and a higher concentration of methylmalonic acid in urine.

**Fig. 2:**
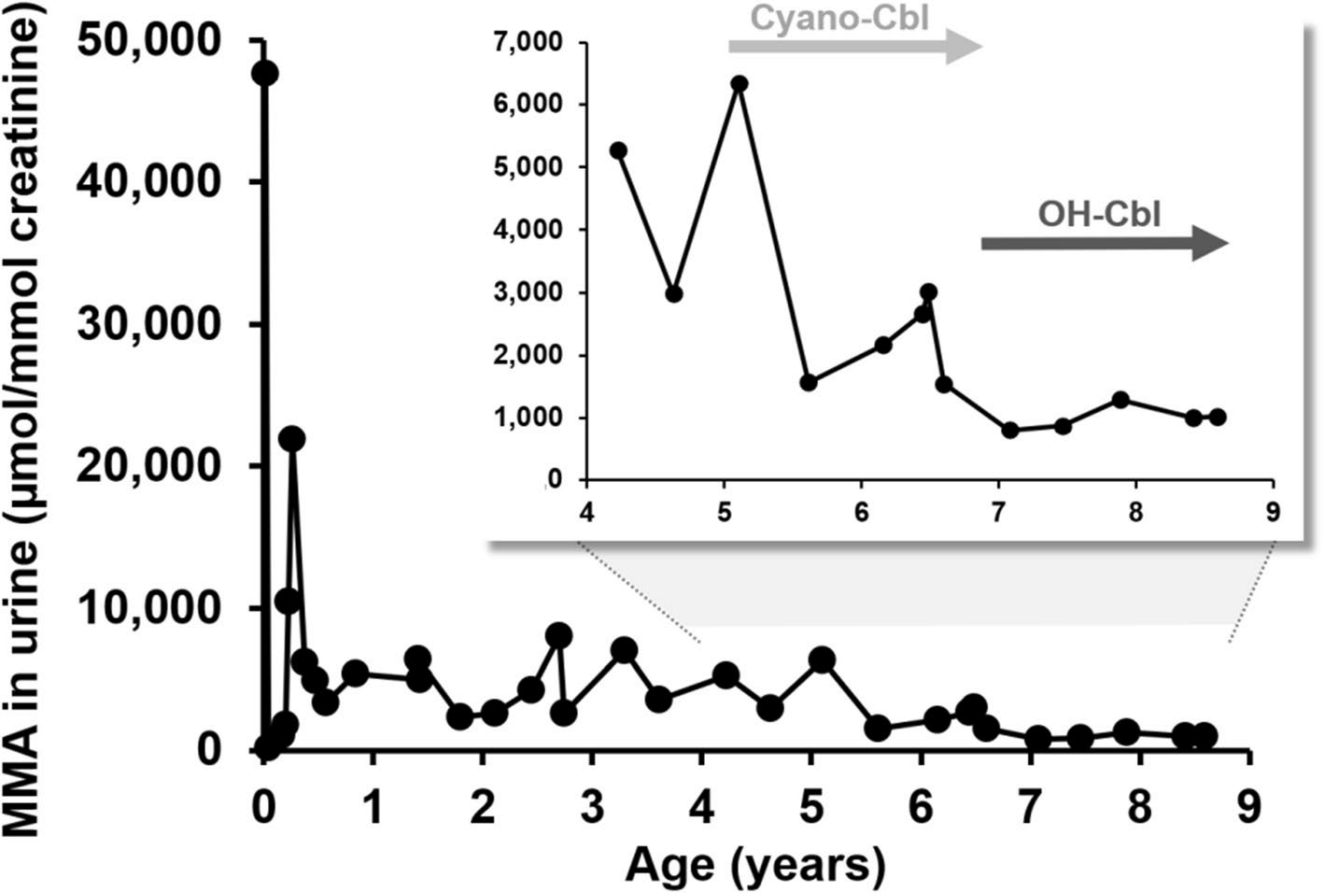
Urinary methylmalonic acid concentration in Patient 2

Sanger sequencing of the *MMAA* gene in the patient’s DNA revealed the presence of the homozygous missense variant NM_172250.3:c.[266T>C];[266T>C], p.[(Leu89Pro)];[(Leu89Pro)]. This variant has been described previously [19, 20] and is classified as pathogenic according to ACMG guidelines [18], with ClinGen Sequence Variant Interpretation Recommendations for PM2 and PM3 (PS4, PM3_strong, PP3, PM2_sup). Based on confirmation of the *CblA* defect by genetic testing in the patient and reports showing the importance of early and adequate vitamin B12 substitution in responsive patients [21], we initiated more intensive i.m. OH-Cbl treatment (1 mg i.m. every week) to prevent renal damage in the future. Currently, the child has an age-appropriate mental development with occasional attention deficit and concentration difficulty at school and is without renal complications. Methylmalonic acid in urine decreased upon the OH-Cbl treatment to 799 – 1,291 μmol/mmol creatinine (Fig. 2).

## Discussion and Conclusions

We present two patients with a severe neonatal manifestation of iMMA who differed in the further clinical course. Based on the genetic diagnosis and current reports [7, 21, 22] we intensified the treatment with Cbl in both patients. Currently, both of them are metabolically stable with better laboratory results.

The first patient was considered as *mut*^*0*^ due to severe neonatal onset and a negative, but at that time not correctly performed, test of cobalamin responsiveness. However, DNA analysis revealed the *CblB* type of iMMA. The variant in the *MMAB* gene found in Patient 1 in a homozygous state, p.Arg186Trp, is usually associated with early onset ranging from 1 day to 18 months [17, 23]. Arg186 is located in the cobalamin-binding pocket of the enzyme [24], and the previously reported propionate incorporation was not increased by the addition of OH-Cbl in the fibroblasts of patients with the biallelic Arg186Trp variant [17, 23]. Nevertheless, intensified treatment of our patient with OH-Cbl resulted in lower methylmalonic acid levels in the urine.

The second patient, with clear Cbl sensitivity, was confirmed as the *CblA* type. The Leu89Pro variant in the *MMAA* gene found in Patient 2 seems to cause severe protein destabilisation [8] corresponding with very early onset of symptoms in our patient, as well as in two other patients reported in the literature [20]. Another patient homozygous for this variant has recently been described with a later onset at 11 months, but with severe neurological complications [19]. Impaired propionate incorporation reported in patient fibroblasts, carrying the Leu89Pro variant in addition to a null variant, clearly showed increased propionate incorporation in the presence of OH-Cbl [20], which is typical for the *CblA* type and in agreement with the cobalamin sensitivity of our Patient 2.

Severe neonatal manifestation of Cbl-responsive iMMA could be misleading in the identification of the iMMA type (Table 1). The severe course is typical for Cbl-nonresponsive forms. Horster et al. showed that 73 % of *mut*^*0*^ patients developed symptoms within the first week of life [7]. Both of our patients had a neonatal manifestation with high excretion of methylmalonic acid in the urine, which is typical for Cbl non-responsive types of iMMA [2, 16]. The Cbl sensitivity test helps to discern between Cbl responsive and non-responsive forms. It is an important part of the diagnostic process, as no child should be given unnecessary or ineffective treatment, especially by injection. The test for early identification of Cbl-sensitive patients should be performed prior to getting the results from enzymological and genetic testing. However, the test of responsiveness is not standardised, and although some recommendations have been made [7, 13, 14], some pitfalls still remain. The Cbl sensitivity test should be performed outside the metabolic crisis and should not be accompanied by the treatment methods as an infusion or extracorporeal elimination, which could influence the test results. The patient should be not receiving cobalamin and be on the same treatment for at least one month [14]. This can be hard to achieve in some patients. Next, the most commonly recommended procedure is 1 mg of OH-Cbl given intramuscularly or intravenously [16]. However, Cbl for parenteral administration is available only in the cyano form in some countries. Furthermore, the recommended duration of the test ranges from 2 days [14] to 1-2 weeks [16]. Here, in the case of Patient 1 (*CblB* type), the neonatologists 19 years ago used only a low dose of cyano-Cbl (0.1 mg instead of 1 mg) due to his young age. This could have led to him initially being evaluated as Cbl-nonresponsive. The additional approach, the *in vitro* Cbl test, is not available in many countries, and even if it is available, the *in vitro* Cbl responsiveness does not reliably predict the *in vivo* responsiveness [25].

**Table 1:** Clinical features of the presented patients compared to the typical iMMA phenotypes

	Cbl-nonresponsive *mut0*, *CblB*	Cbl-responsive *mut*-, *CblA*	Patient 1	Patient 2
Neonatal manifestation	yes	yes	yes, 3^rd^day of life	yes, 4^th^day of life
Severe metabolic acidosis	yes	not typical	yes	yes
Severe hyperammonemia (normal range 0-60 μmol/L)	yes	not typical	yes, 1,094	yes, 1,600
MMA in urine (normal range < 2.5 μmol/mmol creatinine)	1,000-10,000	10-hundreds	32,900	47,686
Severe metabolic crises despite therapy	yes	no	yes	no
Response to the Cbl treatment	no	yes	partial, better to OH-Cbl	yes, better to OH-Cbl
Patient´s working diagnosis			*mut0*	*CblA*, *mut*-
Patient´s confirmed genotype			*MMAB*: c.[556C>T];[556C>T] p.[(Arg186Trp)];[(Arg186Trp)]	*MMAA*: c.[266T>C];[266T>C] p.[(Leu89Pro)];[(Leu89Pro)]

**Fig. 3:**
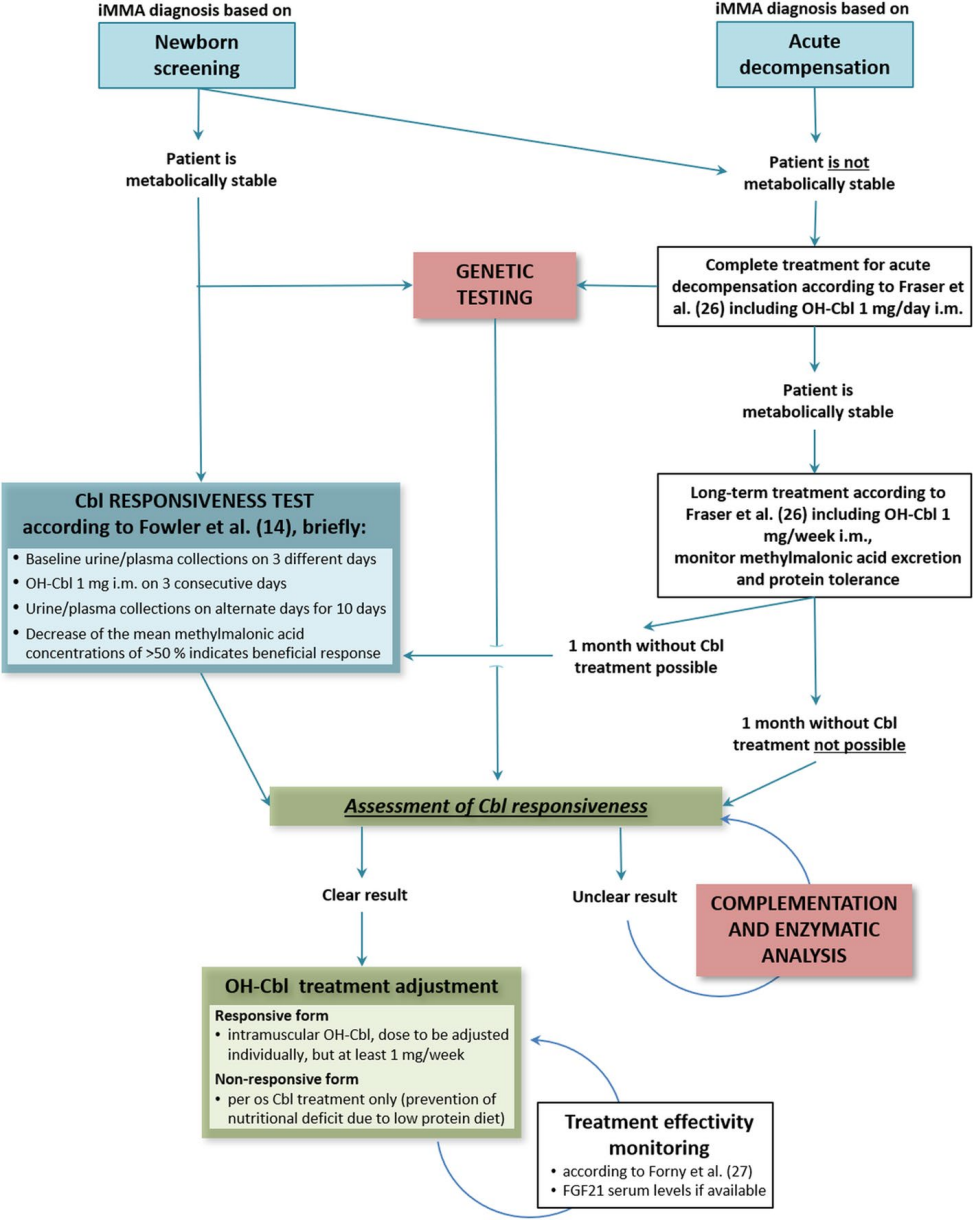
Tentative protocol for the recognition of Cbl sensitive forms of iMMA. Asymptomatic patients or patients stable without vitamin B12 treatment can undergo the test of responsiveness according to Fowler et al [14]. The sensitivity of unstable patients can be deduced from the results of genetic testing or enzymatic studies, if available

Hence, based on our experience, we suggest a scheme of the diagnostic procedure for recognition of Cbl sensitive forms of iMMA (Fig. 3). Fifty-two percent of cobalamin non-responsive and 67 % of responsive MMA cases identified in newborn screening are still asymptomatic at 8 days of life [26] and can be tested as recommended by Fowler et al. [14]. Patients who are not metabolically stable require prior stabilisation, or they may not be suitable for the test at all. In such cases genetic testing is helpful, particularly if enzymatic testing is not available.

It is now accepted that the ideal application route for cobalamin is intramuscular and hydroxocobalamin is the preferred form of the cobalamin molecule [27]. Still, there are various opinions on the dose and frequency of Cbl treatment in Cbl-responsive iMMA patients. Fraser and Venditti [26] suggest 1 mg of OH-Cbl intramuscularly every day in their clinical management guidelines. Intramuscular administration is the main cause of patients’ non-adherence to the treatment; therefore, in clinical practice, it is often necessary to adjust the dose and frequency individually to obtain the best outcome. Our Patient 2 (*CblA* type) had excellent tolerance of protein after the consolidation of clinical status during the neonatal crisis, when the treatment was supported with 1 mg of OH-Cbl every second day. However, due to the poor tolerance of i.m. administration by the child and her mother, it was administered only once per 14 days in the long-term. Nevertheless, after confirming the *CblA* defect by genetic testing, we were able to convince the attending physician and the parents to continue in a more intensive vitamin B12 treatment, as treatment with a higher dose could prevent chronic renal failure in adult age in the Cbl-responsive iMMA type [21]. Similarly, we also intensified the vitamin B12 treatment after confirmation of the *CblB* type in Patient 1. We were also able to switch the cyano-Cbl to OH-Cbl after the genetic confirmation of the diagnosis, as the latter is available for chronic treatment only for patients with confirmed intracellular cobalamin disorder in our country. Acute treatment of patients suspected of having iMMA is available by the parenteral form of OH-Cbl secured by the exceptional import of unregistered therapeutics.

In agreement with other publications [4], we believe that mutation analysis is not only a standard for the diagnosis of iMMA but can also help in selecting a treatment strategy and determining responsiveness to vitamin B12.

The challenge of how to monitor the long-term effectiveness of the therapy to be able to adjust the treatment remains. The serum and urine levels of methylmalonic acid reflect an acute state and can be influenced by renal insufficiency. Serum FGF21 was suggested as a biomarker of long-term complications in organic acidurias, as its levels have high positive and negative predictive value for the occurrence of long-term complications and are not influenced by renal function [28]. New findings in the pathophysiology of organic acidurias show that these defects lead eventually to mitochondrial toxicity. These insights might translate to targeted therapies or predictive biomarkers that could further the development of new algorithms for safe and effective therapies [29].

A limitation of our report was the retrospective evaluation of data and treatment from a period 19 years ago. Patient 1 was the first patient with MMA in our centre, so we had no experience with treatment and follow-up of patients with MMA. Limitations also include an evaluation of methylmalonic acid only in urine in a patient developing chronic renal failure. The plasma methylmalonic acid test has been available in our laboratory only since 2020. Moreover, urinary methylmalonic acid in Patient 1 fluctuated massively over time. This was caused by his frequent metabolic crises which required infusion therapy. Therefore, we selected for the evaluation only the results of methylmalonic acid in urine from outpatient examinations.

The type of iMMA cannot always be correctly assumed based only on the period of clinical manifestation and the excretion of methylmalonic acid in the urine. Our results showed that cobalamin-sensitive patients with iMMA can also present with severe hyperammonaemia and high excretion of MMA in urine in the neonatal period. Similarly, partial responsiveness to Cbl is not always clear, despite performing the *in vivo* sensitivity test. Therefore, early genetic testing, which is more available than *in vitro* Cbl sensitivity tests in many countries, may help to choose the right cobalamin treatment early enough to delay or prevent renal failure.

## Data Availability

All data are available from the corresponding author on reasonable request.

## Abbreviations

Cbl: Cobalamin
i.m.: Intra muscular
iMMA: Isolated methylmalonic aciduria
MMA: Methylmalonic acid
MMCoA: Methylmalonyl-CoA
OH-Cbl: Hydroxocobalamin
tHcy: Total homocysteine
